# Vitamin D as a Lifespan Neuroimmune Signal in Psychiatry: From Developmental Risk to Precision Nutrition

**DOI:** 10.3390/nu18121877

**Published:** 2026-06-10

**Authors:** Czeslaw Ducki, Monika Jach, Michal Pruc, Halla Kaminska, Pawel Pludowski, Lukasz Szarpak

**Affiliations:** 1 Mazovian Specialist Health Centre in Pruszkow, 05-802 Pruszkow, Poland; czeslaw.ducki@mscz.pl; 2 Institute of Biological Sciences, The John Paul II Catholic University of Lublin, 20-708 Lublin, Poland; monika.jach@kul.pl; 3 Institute of Medical Science, The John Paul II Catholic University of Lublin, 20-708 Lublin, Poland; michal.pruc@kul.pl; 4 Department of Children’s Diabetology and Lifestyle Medicine, Faculty of Medical Sciences, Medical University of Silesia, 40-055 Katowice, Poland; 5 Department of Clinical Biochemistry, The Children’s Memorial Health Institute, 04-730 Warsaw, Poland

**Keywords:** ADHD, autism spectrum disorder, depression, neurodevelopment, neuroinflammation, precision nutrition, psychiatry, schizophrenia, vitamin D, vitamin D-binding protein

## Abstract

Background/Objectives: Vitamin D is a nutrient-related secosteroid system with endocrine, paracrine, immunological, and neurodevelopmental actions relevant to nutritional psychiatry. Psychiatric research has often treated vitamin D either as a cross-sectional correlate of depression or as a non-specific supplement expected to act across heterogeneous diagnostic categories. This narrative review aimed to develop a more discriminating framework in which vitamin D is considered a lifespan neuroimmune and immunometabolic signal whose psychiatric relevance depends on developmental timing, biological context, and phenotype. Methods: Evidence was integrated from developmental epidemiology, neonatal dried-blood-spot studies, randomized trials, meta-analyses, Mendelian randomization studies, clinical guidelines, and mechanistic neuroscience. The review focuses on prenatal and neonatal 25-hydroxyvitamin D, vitamin D-binding protein, free and bioavailable vitamin D, vitamin D receptor signaling, immune and microglial pathways, neurotransmitter systems, neurotrophic signaling, mitochondrial function, oxidative stress, hypothalamic–pituitary–adrenal-axis regulation, and the gut–microbiota–immune–brain axis. Results: The available evidence does not support vitamin D as a universal treatment for psychiatric disorders. Instead, vitamin D deficiency and altered vitamin D biology appear most relevant in biologically and clinically defined risk states, including neurodevelopmental vulnerability, inflammatory depression, psychosis liability, severe mental illness with nutritional deprivation, metabolic comorbidity, and cognitive frailty. Mechanistic data support plausible links with cytokine biology, the tryptophan–kynurenine pathway, dopaminergic and serotonergic systems, stress regulation, and neuroimmune homeostasis. Conclusions: Vitamin D should be conceptualized in psychiatry as a context-dependent neuroimmune and immunometabolic signal rather than a generic psychotropic intervention. Future studies should prioritize biomarker-enriched, developmentally timed, nutrition-centered models of precision prevention and adjunctive care.

## 1. Introduction

Vitamin D has classically been discussed in clinical medicine through calcium homeostasis, rickets, osteomalacia, osteoporosis, and fracture prevention [1]. That frame remains clinically essential, but it is too narrow for nutritional neuroscience. Vitamin D is a nutrient-derived secosteroid system generated through cutaneous ultraviolet B exposure and obtained from foods, fortified products, and supplements [2,3]. Its metabolites participate in endocrine and paracrine signaling, immune regulation, cellular differentiation, mitochondrial function, and inflammatory homeostasis [2,4,5,6,7]. In routine practice, serum 25-hydroxyvitamin D (25(OH)D) is the accessible biomarker of vitamin D status, but total 25(OH)D represents only one component of a system that also includes vitamin D-binding protein (DBP), albumin binding, free and bioavailable fractions, vitamin D receptor (VDR) signaling, and enzymatic activation or catabolism [1,8,9,10,11].

Psychiatric disorders are syndromic constructs defined by clinical phenomenology rather than by a single pathobiological mechanism. Major depressive disorder, schizophrenia, bipolar disorder, attention-deficit/hyperactivity disorder (ADHD), autism spectrum disorder (ASD), anxiety disorders, suicidality, and cognitive decline are each biologically heterogeneous. Conversely, inflammatory, metabolic, developmental, and stress-related pathways can cross diagnostic boundaries [12,13,14]. A nutrient-related biomarker such as 25(OH)D is therefore unlikely to map cleanly onto one DSM or ICD diagnosis. A more plausible approach is transdiagnostic and developmental: determine whether vitamin D biology contributes to a broader state of neurodevelopmental or immune-metabolic vulnerability that may be expressed through different psychiatric phenotypes across the lifespan [14,15].

The field has reached an interpretive bottleneck. Observational studies repeatedly associate low 25(OH)D with depressive symptoms, severe mental illness, psychosis, ASD, ADHD, and cognitive impairment, but these findings are vulnerable to reverse causality and confounding [16,17,18]. Psychiatric symptoms may reduce outdoor activity, disrupt diet and sleep, increase adiposity, alter medication exposure, and increase socioeconomic disadvantages, all of which may lower vitamin D status. At the same time, vitamin D deficiency could plausibly affect inflammatory tone, microglial biology, neurotransmission, kynurenine metabolism, neurotrophic signaling, and stress responsivity [6,19,20,21,22]. These explanations are not mutually exclusive. The task for a high-quality review is to separate association from causality, risk modification from symptom response, and clinical correction of deficiency from broad empirical supplementation.

A stronger literature has begun to emerge around early-life exposure. Neonatal dried-blood-spot studies and genetic analyses allow investigators to move closer to the timing of brain development and away from the confounding structure of adult psychiatric illness. The 2025 Danish case–cohort study linking neonatal 25(OH)D and DBP to later neurodevelopmental disorders is especially important because it examines two vitamin D biomarkers and integrates genetic correlates across several psychiatric outcomes [17]. This does not prove that supplementation prevents psychiatric illness, but it materially changes the research agenda: developmental timing and biomarker context are likely more important than adult diagnostic labels alone.

Randomized evidence also requires a more careful reading. Large universal prevention trials in adults, including VITAL-DEP, do not support vitamin D3 supplementation for the prevention of depression in generally sufficient older adults [18]. DFEND, conducted in early psychosis, did not demonstrate clinical benefit despite high rates of deficiency [23]. Conversely, meta-analyses of smaller trials suggest possible short-term symptom improvement in depression, particularly when deficiency, dose, duration, comorbidity, and baseline inflammatory or metabolic vulnerability are considered [24,25,26]. These data are not contradictory if interpreted through a precision nutrition framework: unselected supplementation is a weak test of a biologically stratified hypothesis. To avoid overloading the narrative sections with trial-specific estimates, key quantitative findings from pivotal randomized and developmental vitamin D studies relevant to psychiatry are summarized in Supplementary Table S1.

The relevant clinical questions include diet quality, fortification, sunlight exposure, deficiency, supplementation safety, assay standardization, high-risk populations, and the integration of nutritional assessment into psychiatric care. The most useful contribution is therefore neither a general review of vitamin D and depression nor a catalogue of positive associations. It is an evidence-calibrated model that treats vitamin D as a modifiable nutritional exposure, a biomarker of biological context, and a candidate precision prevention target.

The aim of this narrative review is to synthesize the evidence linking vitamin D biology with psychiatric risk and outcomes across the lifespan; to distinguish developmental, mechanistic, observational, and interventional evidence; and to propose a biomarker-informed framework for future clinical nutrition research and psychiatric practice.

## 2. Methods: Literature Search Strategy for a Narrative Review

This article is a narrative and integrative review, not a systematic review and not a meta-analysis. It was designed to synthesize biologically and clinically relevant evidence across psychiatry, nutritional neuroscience, clinical nutrition, developmental epidemiology, immunometabolism, and preventive medicine. The review does not provide pooled effect estimates and does not claim PRISMA-compliant systematic coverage of all eligible studies.

The literature was searched in PubMed/MEDLINE, Scopus, Web of Science, Embase, PsycINFO, the Cochrane Library, and Google Scholar for citation chaining, from database inception to 9 May 2026. Search terms combined vitamin D, 25-hydroxyvitamin D, cholecalciferol, vitamin D-binding protein, DBP, VDR, free vitamin D, bioavailable vitamin D, depression, major depressive disorder, schizophrenia, psychosis, bipolar disorder, ADHD, autism spectrum disorder, anxiety, suicidality, cognition, severe mental illness, neuroinflammation, kynurenine, tryptophan, prenatal, neonatal, neurodevelopment, supplementation, randomized trial, cohort, Mendelian randomization, meta-analysis, and guideline.

Priority was given to peer-reviewed human studies, randomized controlled trials, systematic reviews, meta-analyses, umbrella reviews, population-based cohorts, birth-cohort and neonatal dried-blood-spot studies, Mendelian randomization analyses, and major clinical guidelines. Mechanistic and preclinical studies were included selectively when they clarified plausible biological pathways not directly measurable in humans. Low-quality, duplicated, non-peer-reviewed, purely speculative, or clinically irrelevant sources were not used as primary evidence.

Study selection was purposive and iterative rather than checklist-based. Records were first screened for relevance to vitamin D biology, psychiatric or neurodevelopmental outcomes, nutritional status, biomarker interpretation, or clinical applicability. When several sources addressed the same question, priority was given to recent systematic reviews or meta-analyses, large population-based cohorts, randomized trials, Mendelian randomization studies, neonatal or birth-cohort designs, and clinical guidelines; smaller observational or mechanistic studies were retained when they clarified phenotype-specific or pathway-specific questions not covered by higher-level evidence.

Evidence was interpreted using an evidence-calibrated hierarchy. Randomized trials and clinical guidelines were weighted most heavily for supplementation and practice recommendations; birth-cohort, neonatal, and genetic studies were weighted heavily for developmental hypotheses; observational adult psychiatric studies were treated as hypothesis-generating unless temporality and confounding were addressed; and mechanistic studies were used to explain plausibility rather than to infer clinical efficacy. The evidence-calibration framework used to structure the search strategy, source prioritization, and interpretation of findings is summarized in Table 1.

Throughout the review, terms are used deliberately. Association denotes statistical co-occurrence; risk modification denotes evidence compatible with altered future disease probability; treatment response denotes symptom change after intervention; and prevention denotes reduction in incident disease. These categories are kept separate because much of the vitamin D-psychiatry literature loses clinical precision by conflating them.

## 3. Vitamin D Biology: Why Total 25(OH)D Is Necessary but Not Sufficient

The biological argument for vitamin D in psychiatry is credible only if the exposure is defined accurately. Total serum 25(OH)D is clinically useful, but psychiatric studies that rely on it alone may misclassify the relevant biology, particularly in pregnancy, inflammation, obesity, severe mental illness, liver disease, and genetically determined variation in vitamin D-binding protein (DBP) [10,11,27]. A top-tier research agenda must therefore move from a single-analyte model toward a vitamin D system model.

**Figure 1:**
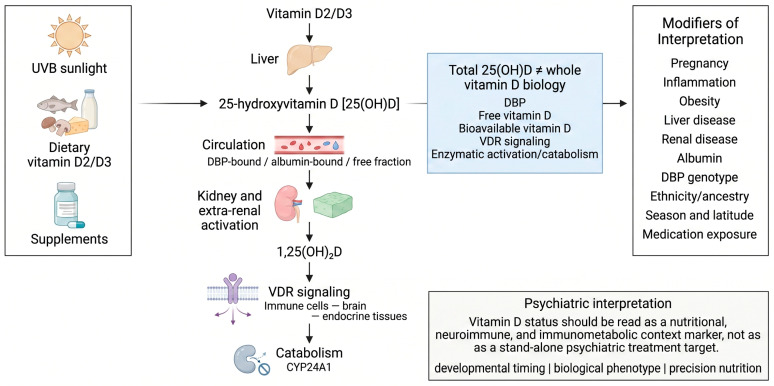
Vitamin D biology and biomarker interpretation in psychiatric populations. Vitamin D status reflects cutaneous UVB exposure, dietary vitamin D_2_/D_3_, fortified foods, and supplementation. Vitamin D is converted to 25-hydroxyvitamin D [25(OH)D], circulates when mainly bound to vitamin D-binding protein (DBP) and albumin, and undergoes renal and extra-renal activation to 1,25(OH)_2_D, followed by VDR-mediated signaling and CYP24A1-dependent catabolism. Total 25(OH)D is clinically useful but does not capture the whole vitamin D system. Interpretation may be modified by pregnancy, inflammation, obesity, liver disease, renal disease, albumin concentration, DBP genotype, ancestry, season, latitude, and medication exposure. In psychiatry, vitamin D status should therefore be read as a nutritional, neuroimmune, and immunometabolic context marker rather than as a stand-alone psychiatric treatment target.

Within the vitamin D system, cutaneous ultraviolet B exposure, diet, fortified foods, and supplements provide vitamin D_2_ and vitamin D_3_. These metabolites circulate predominantly bound to vitamin DBP and albumin, are hydroxylated in the liver to 25(OH)D, and are then converted by renal and extra-renal 1α-hydroxylase to 1,25(OH)_2_D, which signals through the VDR and is catabolized through CYP24A1-dependent pathways [8,11,28,29,30]. VDR and vitamin D-activating enzymes have been identified in human brain tissue and immune cells, including regions and pathways relevant to mood, cognition, reward, psychosis vulnerability, neurotrophic signaling, oxidative stress, synaptic maturation, and cellular energetics [29,30,31,32,33,34]. Total 25(OH)D remains the most practical clinical biomarker, but it is an incomplete proxy for biologically available vitamin D because most circulating vitamin D metabolites are protein-bound and because DBP concentration and polymorphisms, albumin, pregnancy, inflammation, obesity, liver and renal disease, ancestry, season, latitude, and medication exposure may modify interpretation [1,8,9,10,11,27]. This is particularly relevant in perinatal psychiatry, severe mental illness, inflammatory depression, frailty, and medically complex psychiatric populations [1,10,11,17]. The core metabolic and biomarker relationships are summarized in Figure 1; subsequent sections therefore focus on psychiatric interpretation rather than repeating the metabolic pathway.

At the same time, the clinical translation of free and bioavailable vitamin D remains limited by incomplete assay standardization and uneven availability across healthcare settings. Direct free 25(OH)D assays, calculated free vitamin D estimates, DBP measurement, albumin correction, and genotype-sensitive interpretation are not interchangeable, and many psychiatric services do not have routine access to validated free or bioavailable vitamin D testing. These measures should therefore be regarded primarily as research-level enrichment variables or specialist adjuncts, rather than as prerequisites for ordinary clinical correction of vitamin D deficiency [10,11].

Genetic determinants of vitamin D status include variants in genes involved in synthesis, transport, activation, catabolism, and receptor signaling, including GC, VDR, CYP2R1, CYP27B1, CYP24A1, and DHCR7/NADSYN1 [35,36,37,38,39]. Genome-wide association studies have identified multiple loci associated with circulating 25(OH)D, and neonatal dried-blood-spot work has extended genetic inquiry into DBP and early-life vitamin D biology [36,38,40]. These data are valuable because they reduce, though do not eliminate, some confounding that limits ordinary observational studies.

Clinically, vitamin D status is shaped by nutritional and environmental determinants: dietary intake, fortified foods, supplement use, ultraviolet B exposure, latitude, season, air pollution, clothing, skin pigmentation, age, obesity, pregnancy, lactation, malabsorption, renal or liver disease, and medication use [8,41,42,43,44]. Psychiatric care adds further risk: reduced outdoor activity during depressive episodes, negative symptoms or institutionalization in psychosis, antipsychotic-associated weight gain, substance use, social deprivation, and reduced preventive medical care. For these reasons, vitamin D status in psychiatric cohorts should be interpreted as both a nutritional biomarker and a possible marker of illness ecology.

## 4. Why Psychiatry Needs a Lifespan Vitamin D Framework

The central timing problem is simple but often ignored: vitamin D insufficiency in late adulthood is not biologically equivalent to low vitamin D status during fetal brain development [34,44,45,46]. The same biomarker may indicate developmental exposure, a lifestyle correlate of illness, a metabolic risk state, or a treatment target depending on age and context.

The lifespan framework begins with timing. Vitamin D deficiency during fetal brain development is not equivalent to vitamin D deficiency in a 45-year-old patient with recurrent depression or a 78-year-old patient with frailty and cognitive symptoms. Prenatal and neonatal periods involve neurogenesis, neuronal migration, differentiation, synaptogenesis, early dopaminergic organization, immune programming, and placental-fetal signaling [34,46,47,48]. Childhood and adolescence involve immune maturation, synaptic pruning, hormonal transition, stress-system calibration, and emergence of neurodevelopmental and affective symptoms. Adult and later-life states involve chronic inflammation, metabolic disease, vascular risk, medication exposure, and neurodegeneration. The same biomarker can therefore have different meaning across time [44,45,49].

Developmental vitamin D research is clinically compelling because early-life exposure precedes psychiatric diagnosis, reducing reverse causality. Systematic reviews of maternal and perinatal vitamin D status suggest a small but biologically coherent body of evidence linking low prenatal or neonatal vitamin D with ASD, ADHD, and schizophrenia, although heterogeneity remains considerable and causality is not established [45]. Stronger signals tend to arise from larger registry-based cohorts, stricter outcome definitions, and settings where neonatal samples allow temporally informative biomarker measurement [17,50].

Schizophrenia provides the historical prototype. Ecological and epidemiological observations—season of birth, latitude, migration, urbanicity, and prenatal famine—were never specific to vitamin D, but they contributed to the developmental vitamin D deficiency hypothesis [50,51]. Neonatal dried-blood-spot studies subsequently found associations between low neonatal 25(OH)D and later schizophrenia, and later research strengthened the link by incorporating larger samples and better covariate control [50,52,53]. These findings remain probabilistic: most neonates with low 25(OH)D will not develop psychosis, and psychosis is not reducible to vitamin D status [50,51].

ADHD and ASD extend the model beyond psychosis. Maternal and neonatal studies show associations between lower vitamin D status and later ADHD or ASD diagnosis or traits [17,45,54,55,56,57]. The strongest interpretation is risk modulation in susceptible developmental contexts, not simple causation. A randomized pregnancy trial did not show that high-dose supplementation from mid-pregnancy prevents autism or ADHD at age 10, although higher pre-intervention 25(OH)D was associated with lower risk [54]. This distinction is crucial: observational associations can be real while late, non-stratified, or inadequately targeted supplementation fails to alter long-term outcomes [54,58].

The lifespan model must also separate several clinical questions. Does vitamin D modify liability before disease onset? Does deficiency influence symptom expression after disease onset? Does correction of deficiency improve treatment response? Does supplementation prevent relapse or reduce disability? These are different questions requiring different designs. Perinatal prevention studies need long follow-up and developmental endpoints [1,54]. Depression trials need baseline deficiency, inflammatory phenotype, and adequate duration. Severe mental illness studies need physical-health outcomes, adherence, and safety monitoring. A single generic supplementation trial cannot answer all these questions.

## 5. Mechanistic Convergence: Neuroimmune and Immunometabolic Pathways

Mechanistic evidence should be interpreted as converging biological plausibility, not as proof of causality [4,6,7,19,32,59,60]. The most clinically credible argument for vitamin D in psychiatry is not that one molecular pathway explains one psychiatric disorder. Rather, vitamin D biology intersects with several systems that clinicians repeatedly encounter in patients with psychiatric illness: immune activation, metabolic dysfunction, oxidative stress, fatigue, cognitive inefficiency, sleep disturbance, endocrine dysregulation, poor diet quality, low sunlight exposure, obesity, frailty, and chronic medical comorbidity [3,61,62]. The question is therefore not whether vitamin D is a psychiatric treatment, but whether vitamin D status helps identify biologically vulnerable subgroups in whom nutritional correction is clinically meaningful [19,20,63]. The major mechanistic domains linking vitamin D biology with psychiatric phenotypes are summarized in Table 2, with emphasis on translational interpretation rather than causal overstatement.

**Figure 2:**
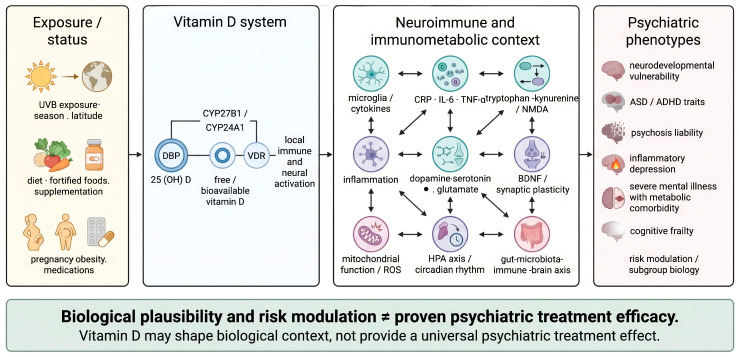
Vitamin D as a lifespan neuroimmune and immunometabolic signal in psychiatry. Environmental, nutritional, physiological, and treatment-related determinants shape vitamin D exposure and status. Total 25(OH)D, DBP/free or bioavailable vitamin D, enzymatic activation/catabolism, and VDR signaling may intersect with inflammatory, neuroimmune, metabolic, neurotransmitter, neurotrophic, mitochondrial, HPA-axis, circadian, and gut–microbiota–immune–brain pathways. These mechanisms may contribute to risk modulation or subgroup biology across neurodevelopmental vulnerability, ASD/ADHD traits, psychosis liability, inflammatory depression, severe mental illness with metabolic comorbidity, and cognitive frailty. The figure does not imply that vitamin D is a stand-alone psychiatric treatment.

Figure 2 provides the conceptual framework of the review, linking vitamin D exposure and biomarker status with vitamin D system biology, neuroimmune and immunometabolic pathways, and selected psychiatric risk phenotypes, while distinguishing biological plausibility from proven psychiatric treatment efficacy.

At the neuroimmune level, vitamin D biology is most relevant as a regulator of immune tone and microglial activity. VDR expression in immune cells and local conversion of 25(OH)D to 1,25(OH)_2_D support paracrine and autocrine effects on antigen presentation, T-cell differentiation, regulatory T-cell activity, macrophage function, cytokine production, and microglial activation [3,6,59,60,61,62,63,64,65,66,67]. In clinical psychiatry, these pathways are most plausibly relevant to inflammatory phenotypes characterized by anergia, psychomotor slowing, sleep disturbance, pain sensitivity, cognitive dulling, anhedonia, metabolic deterioration, and poor treatment response across depression, bipolar disorder, psychosis, post-infectious states, obesity-associated mood disturbance, and older-age presentations [68,69,70]. Vitamin D should therefore be read within the broader nutritional and endocrine background that may shape inflammatory vulnerability [11,67]. Microglia provide a developmental bridge because they participate in synaptic pruning, myelination, neurogenesis, circuit refinement, and response to injury; this is particularly relevant during fetal life, infancy, childhood, and adolescence, when immune and neural developmental programs are closely coupled [17,19,20,21]. These mechanisms should still be interpreted as stratification pathways rather than direct treatment mechanisms.

**Figure 3:**
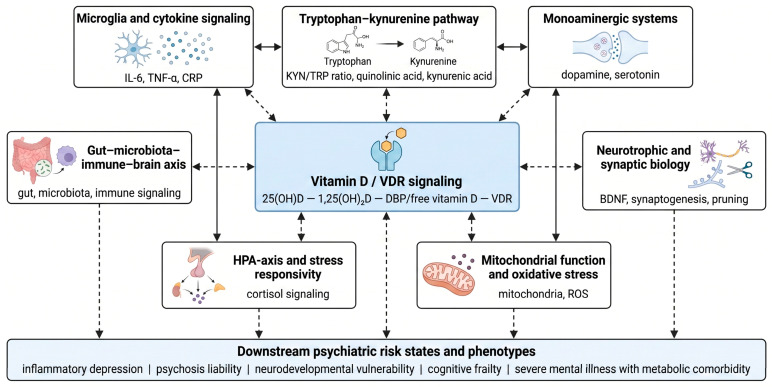
Mechanistic convergence of vitamin D/VDR signaling with neuroimmune and immunometabolic pathways relevant to psychiatric risk phenotypes. Vitamin D/VDR signaling, including total 25(OH)D, 1,25(OH)_2_D, DBP/free vitamin D, and VDR activity, may intersect with microglial and cytokine signaling, the tryptophan–kynurenine pathway, monoaminergic systems, neurotrophic and synaptic biology, mitochondrial function and oxidative stress, HPA-axis and stress responsivity, and the gut–microbiota–immune–brain axis. These pathways provide biological plausibility for links with inflammatory depression, psychosis liability, neurodevelopmental vulnerability, cognitive frailty, and severe mental illness with metabolic comorbidity. The figure represents mechanistic plausibility and risk-state biology, not established psychiatric treatment efficacy.

The clinical interpretation should remain cautious. Low vitamin D status during sensitive developmental windows may plausibly lower the threshold for maladaptive neuroimmune responses in genetically or environmentally susceptible individuals [17,70]. That hypothesis is compatible with the developmental literature on schizophrenia, ASD, ADHD, and later affective vulnerability. It does not mean that vitamin D deficiency causes these disorders. It means that vitamin D may be one modifiable component within a broader developmental risk architecture [71,72]. The mechanistic convergence of vitamin D/VDR signaling with neuroimmune, neurotransmitter, neurotrophic, mitochondrial, stress-system, and gut–brain pathways is summarized in Figure 3.

The tryptophan–kynurenine pathway is one of the most persuasive mechanistic intersections. Pro-inflammatory cytokines can shift tryptophan metabolism away from serotonin synthesis and toward kynurenine metabolites [73,74,75]. Several of these metabolites have neuroactive properties, including effects on glutamatergic signaling, oxidative stress, excitotoxicity, sleep, cognition, and affective regulation [25,26,27]. This pathway has been implicated in depression, suicidality, psychosis, cognitive impairment, and sickness-behavior phenotypes [74,75].

Vitamin D may influence this pathway indirectly through immune regulation and possibly through effects on enzymes involved in tryptophan and serotonin biology [73,76]. The practical implication is not that serum 25(OH)D is a serotonin marker. A more clinically defensible interpretation is that low 25(OH)D in an inflamed, obese, frail, or nutritionally depleted patient may signal a biological environment in which tryptophan metabolism, inflammatory tone, and neurobehavioral symptoms are moving in the same adverse direction [77,78].

Vitamin D also intersects with neurotransmitter and neurotrophic systems. It has been linked to dopaminergic development, catecholamine biology, serotonergic regulation, and pathways influencing glutamatergic and GABAergic balance [19,22,32]. These mechanisms are relevant to reward processing, motivation, attentional phenotypes, psychosis liability, and affective symptoms [29]. They should not, however, be overread as direct antidepressant or antipsychotic mechanisms. Similarly, vitamin D may support neurotrophic signaling, neuronal differentiation, and synaptic plasticity, including pathways involving brain-derived neurotrophic factor and related systems [79,80].

Mitochondrial function and oxidative stress add another clinically recognizable layer. Many psychiatric patients do not present with mood symptoms alone. They present with fatigue, reduced exercise tolerance, psychomotor slowing, sleep disruption, pain, cognitive inefficiency, insulin resistance, weight gain, and systemic inflammation [81,82]. Vitamin D has been linked experimentally to mitochondrial energetics, antioxidant defense, calcium handling, and cellular resilience [83,84]. Deficiency may therefore reduce physiological reserve in patients already exposed to inflammatory, metabolic, pharmacological, or psychosocial stressors [85,86].

Immunometabolism is probably where this biology becomes most relevant to day-to-day psychiatric care. Obesity, insulin resistance, dyslipidemia, hepatic steatosis, low physical activity, and antipsychotic-associated weight gain are common in psychiatric populations and all complicate interpretation of total 25(OH)D [10]. Low vitamin D status in obesity may reflect volumetric dilution, altered adipose storage, reduced outdoor activity, poor diet quality, and chronic inflammation [8,86]. At the same time, vitamin D biology intersects with adipocyte function, insulin signaling, pancreatic beta-cell physiology, and inflammatory mediators [81,86]. Thus, in depression with obesity or schizophrenia with metabolic syndrome, low vitamin D may be both a nutritional abnormality and a marker of broader immunometabolic risk [10,67].

The gut–microbiota–immune–brain axis provides a further plausible link, though one that requires disciplined interpretation. Vitamin D contributes to epithelial barrier integrity, antimicrobial peptide expression, mucosal immune regulation, and host-microbiome interactions [3,59,62]. Altered gut permeability and immune signaling may influence systemic inflammation, tryptophan metabolism, vagal pathways, and neuroactive metabolites [19,73]. Given the frequency of gastrointestinal symptoms, restrictive diets, obesity, inflammation, and psychotropic medication exposure in psychiatric patients, this pathway is clinically relevant, but still not sufficient to infer treatment efficacy [19,78].

Overall, these pathways support biological plausibility and help identify clinically recognizable risk phenotypes, including prenatal or neonatal vulnerability, inflammatory depression, early psychosis with nutritional deprivation, severe mental illness with metabolic comorbidity, older-age frailty, and chronic illness with poor diet or limited sunlight exposure [51,87,88].

Because obesity, low-grade inflammation, poor diet quality, limited sunlight exposure, metabolic comorbidity, and multimorbidity recur across psychiatric populations, we treat these factors as cross-cutting modifiers rather than reintroducing them in detail in every disorder-specific section. Subsequent sections therefore focus on what is distinctive for each phenotype: timing, strength of evidence, direction of causality, and clinical interpretability.

## 6. Depressive Disorders: Separating Treatment Signal from Prevention Signal

Depression is the largest clinical literature on vitamin D, and precisely for that reason it requires strict separation of three questions: whether vitamin D prevents depression incidents, whether supplementation modifies symptoms in established depression, and whether correcting deficiency improves general nutritional and medical status in depressed patients [26,89]. Collapsing these questions is the main source of overstatement in the field.

Observational studies and meta-analyses consistently link lower 25(OH)D with depressive symptoms, but the direction of effect remains uncertain [16,89,90]. Depressive illness can reduce sunlight exposure, physical activity, appetite, diet quality, sleep, and social participation; it also clusters with obesity, inflammation, socioeconomic adversity, and medical comorbidity [16,91]. Low 25(OH)D may therefore be causal, consequential, epiphenomenal, or part of a bidirectional illness-nutrition loop [16,89,90].

Randomized evidence is mixed but informative. VITAL-DEP randomized 18,353 adults aged 50 years or older without clinically relevant depressive symptoms at baseline to vitamin D3 2000 IU/day or placebo for a median of 5.3 years. Depression or clinically relevant depressive symptoms occurred in 609 participants in the vitamin D group and 625 in the placebo group, corresponding to 12.9 vs. 13.3 events per 1000 person-years; the hazard ratio was 0.97 (95% CI, 0.87–1.09; *p* = 0.62). Change in PHQ-8 mood score was also null, with a mean difference of 0.01 points (95% CI, −0.04 to 0.05). The mean baseline 25(OH)D concentration in the parent VITAL trial was 30.8 ng/mL, suggesting that this was not a deficiency-enriched trial [18]. Thus, VITAL-DEP should be interpreted primarily as evidence against universal depression prevention in a generally vitamin D-sufficient older adult population, rather than as a definitive test of whether correction of deficiency may improve depressive symptoms in biologically enriched subgroups. In contrast, recent meta-analyses of randomized trials report heterogeneous short-term reductions in depressive symptom scores, particularly in symptomatic or deficient participants; however, these signals remain inconsistent and should be interpreted as hypothesis-generating rather than as evidence of a clear antidepressant effect [24,25,26,92]. A compact quantitative summary of these and other pivotal intervention or developmental studies, including vitamin D dose, treatment duration, baseline or achieved 25(OH)D where reported, and primary effect estimates, is provided in Supplementary Table S1.

In depression, these cross-cutting determinants are clinically relevant mainly as effect modifiers. Low 25(OH)D should be interpreted alongside adiposity, inflammatory status, diet quality, season, sunlight exposure, and medical comorbidity, because these variables may explain why universal prevention trials are negative while deficiency-enriched or symptom-focused trials sometimes show a short-term signal [24,26,92].

The most defensible clinical position is precision-oriented but conservative. Vitamin D should not be promoted as monotherapy for major depressive disorder or as an intervention with established antidepressant efficacy [93]. It may be reasonable to assess and correct deficiency according to general medical and nutritional indications, particularly in clinically vulnerable or nutritionally at-risk patients [94]. For psychiatric research, the priority is to test whether correction of deficiency as part of adjunctive nutritional care has clinically meaningful effects in deficient, inflamed, metabolically vulnerable, or nutritionally compromised subgroups [95]. Trials should stratify by baseline 25(OH)D, DBP/free vitamin D, CRP, BMI, sex, antidepressant treatment, season, adherence, achieved serum response, and safety [11,95].

## 7. Schizophrenia and Psychosis: Developmental Liability More than Late Symptom Treatment

Psychosis is the clearest stress test for the lifespan model because the strongest vitamin D signal lies upstream of clinical onset, whereas trials in established illness have been less persuasive [50,51]. A negative adult supplementation trial should therefore not be interpreted as evidence against a developmental prevention hypothesis.

The developmental vitamin D deficiency hypothesis proposes that early-life deficiency may influence brain development, dopaminergic maturation, immune priming, and later psychosis vulnerability [17,50,51]. Neonatal and developmental studies support a probabilistic association when vitamin D status is measured before disease onset, although the effect is neither deterministic nor specific to schizophrenia [17,50,52].

In psychosis, adult vitamin D deficiency is best interpreted through illness ecology and physical-health risk, whereas the stronger etiological hypothesis concerns prenatal or neonatal exposure [96]. Thus, low 25(OH)D in established psychosis should not be overread as a direct antipsychotic target, but it remains clinically relevant for nutritional, cardiometabolic, and skeletal health [97,98,99,100].

The DFEND randomized clinical trial assigned 149 adults with early psychosis to monthly cholecalciferol 120,000 IU or placebo for 6 months. Baseline mean 25(OH)D was low in both groups: 14.30 ng/mL in the vitamin D arm and 15.93 ng/mL in the placebo arm; 74.6% of participants had concentrations below 20 ng/mL. At 6 months, 25(OH)D increased to 32.97 ng/mL in the vitamin D arm and remained 15.89 ng/mL in the placebo arm, but the primary psychiatric outcome was unchanged: PANSS total score mean difference 3.57 (95% CI, −1.11 to 8.25; *p* = 0.13) [23]. Importantly, the trial also documented very high rates of vitamin D insufficiency, particularly among Black and other minoritized participants, demonstrating a major public-health issue even when psychiatric symptom benefit is absent [23]. This distinction reinforces the need to separate psychiatric efficacy from broader nutritional and physical-health indications. Accordingly, DFEND argues against assuming psychiatric symptom improvement from biochemical correction of vitamin D deficiency in early psychosis, but it does not negate the developmental-risk hypothesis or the need to identify and treat deficiency for established medical and nutritional indications.

Future psychosis studies should be more targeted. Perinatal studies should test whether early vitamin D optimization modifies later neurodevelopmental and psychosis risk, recognizing ethical and logistical complexity [17]. Early psychosis trials should focus on patients with confirmed deficiency, measure DBP/free vitamin D and inflammatory/metabolic biomarkers, and include negative symptoms, cognition, functioning, cardiometabolic outcomes, and safety [97,100]. Chronic schizophrenia studies should address institutional nutrition, obesity, metabolic syndrome, falls, bone health, and general medical morbidity.

## 8. Vitamin D, ADHD, and Autism Spectrum Disorder: Probabilistic Risk Modulation

For ADHD and ASD, the vitamin D question is primarily developmental. The key issue is not whether vitamin D explains these heterogeneous conditions, but whether prenatal or neonatal vitamin D biology modifies risk probability in susceptible developmental contexts [17,45,54].

ADHD and ASD are central to the developmental vitamin D model because they typically emerge early, have strong neurodevelopmental components, and show immune, genetic, environmental, and metabolic heterogeneity [17,45]. Maternal and neonatal 25(OH)D studies have reported associations with ADHD and ASD diagnoses or traits, including large register-based studies and the Stockholm Youth Cohort [17,55,56,57]. These findings suggest that early vitamin D status may contribute to neurodevelopmental risk architecture, but they do not establish deterministic causation [17,45,54].

These mechanisms are not disorder-specific; they support developmental plausibility but do not justify deterministic causal or therapeutic claims. ADHD and ASD remain heterogeneous conditions shaped by genetic liability, prenatal exposures, perinatal complications, family history, environmental context, and social determinants [34,48].

Intervention data are less definitive than observational data. Pediatric ASD supplementation trials and meta-analyses have suggested possible improvements in some behavioral domains, such as stereotyped behavior, but effects are inconsistent and study quality is variable. In the COPSAC/COPYCH pregnancy trial, 623 mothers were randomized to high-dose vitamin D3 2800 IU/day or standard-dose 400 IU/day from gestational week 24 until 1 week postpartum. At age 10, high-dose supplementation was not associated with autism or ADHD risk. However, higher maternal preintervention 25(OH)D was associated with lower risk of autism (OR per 10 nmol/L, 0.76; 95% CI, 0.59–0.97; *p* = 0.034), lower autistic symptom load (β per 10 nmol/L, −0.03; 95% CI, −0.05 to 0.00; *p* = 0.024), and lower risk of ADHD diagnosis (OR per 10 nmol/L, 0.88; 95% CI, 0.78–0.99; *p* = 0.033) [54]. This supports a refined interpretation: timing, baseline status, dose, duration, and biological susceptibility matter [54,58].

The clinical message is narrow but important. Vitamin D sufficiency in pregnancy, lactation, infancy, and childhood is already relevant for general health [1]. The psychiatric question is whether certain neurodevelopmental risk groups benefit from optimized nutritional status beyond standard care. Future studies should avoid language implying that vitamin D causes or prevents ASD/ADHD; instead, they should test whether vitamin D biology modifies risk probability, symptom burden, or developmental trajectories in well-characterized subgroups.

## 9. Bipolar Disorder, Anxiety, Suicidality, Cognition, and Severe Mental Illness

Across these domains, evidence is not strong enough to support disorder-specific therapeutic claims. Vitamin D is more defensibly interpreted as a marker of broader nutritional, inflammatory, metabolic, and frailty-related vulnerability, particularly in patients with severe mental illness or multimorbidity [73,101,102,103,104,105,106,107,108,109,110,111,112,113,114,115,116].

Bipolar disorder has a smaller and more heterogeneous vitamin D literature than depression. Some studies report low 25(OH)D or associations with illness phase, mood instability, inflammatory state, or metabolic comorbidity, but evidence is insufficient to recommend vitamin D as a mood-stabilizing treatment [73,101]. The more clinically grounded interpretation is that bipolar disorder carries risk for nutritional deficiency through reduced routine, seasonal mood variation, obesity, medication burden, hospitalization, and physical inactivity [101,102,103].

Anxiety disorders and stress-related phenotypes have been examined less rigorously. Some trials and meta-analyses include anxiety outcomes, but recent dose–response depression work has not shown a consistent anxiety effect [24]. Anxiety-related associations may be mediated by sleep, inflammation, endocrine status, or lifestyle rather than vitamin D-specific pathways [72,104]. Future trials should not use anxiety as a secondary endpoint without adequate power and phenotype definition [24,105].

Suicidality deserves a cautious but more explicit interpretation. Observational syntheses and case–control studies have reported associations between low vitamin D status and suicidal ideation, suicide attempts, or suicide risk, plausibly through shared links with depression severity, immune activation, pain, sleep disruption, impulsivity, social adversity, and medical comorbidity [106,107,108,109,110,111]. However, these pathways are clinically non-specific and highly vulnerable to reverse causality. In a suicidal patient, low 25(OH)D may reflect severe depressive withdrawal, reduced outdoor exposure, poor diet, substance use, chronic inflammation, physical illness, or socioeconomic deprivation rather than a causal pathway to suicidal behavior. Vitamin D assessment and correction may be appropriate when general deficiency risk is present, but supplementation should not be framed as suicide prevention. The clinical response to suicidality remains urgent psychiatric risk assessment, safety planning, evidence-based treatment of the underlying disorder, and correction of broader nutritional and medical vulnerability.

Cognition and older-age psychiatric presentations require separate consideration. Vitamin D status has been associated with cognitive function and frailty in observational studies, but randomized supplementation has not reliably prevented cognitive decline in generally replete older adults [112,113]. The more plausible clinical use is targeted correction of deficiency in older psychiatric patients with frailty, falls risk, poor diet, institutionalization, sarcopenia, or multimorbidity, integrated with exercise, protein adequacy, medication review, sleep, and vascular risk management [114,115,116].

Severe mental illness warrants special emphasis because it concentrates several vitamin D risk pathways in the same patients: reduced outdoor activity, negative symptoms, poverty, smoking, obesity, poor diet, fragmented preventive care, institutional exposure, and antipsychotic-associated metabolic syndrome [51,87]. Assessment in this group is therefore best integrated into routine physical-health and nutritional care [99].

## 10. Vitamin D as a Transdiagnostic Biomarker in Psychiatry

The next generation of studies should be phenotype-first. Vitamin D biology is most likely to matter when it marks a coherent nutritional, inflammatory, metabolic, developmental, or frailty-related subgroup rather than a broad DSM/ICD diagnosis [64,65,117].

Examples make this point clinically concrete: a patient with depression, obesity, elevated CRP, poor diet, low 25(OH)D, and antipsychotic exposure has a different vitamin D problem from a child with ASD risk, prenatal deficiency, and maternal inflammation, or from a first-episode psychosis patient with low DBP, low total 25(OH)D, and high metabolic risk [67,118]. The diagnosis is relevant, but insufficient for deciding whether vitamin D assessment or supplementation is biologically meaningful [11,17,71].

Accordingly, candidate vitamin D-responsive or vitamin D-informative phenotypes include inflammatory depression, neurodevelopmental risk in the prenatal to early-childhood window, metabolic syndrome-associated psychiatric illness, cognitive impairment or frailty in older adults, severe mental illness with nutritional deprivation, and perinatal risk states [65,67,72,118]. They should be treated as rational targets for stratified research and clinically sensible nutritional assessment [10,72,118]. A biomarker-informed precision framework for identifying vitamin D-informative psychiatric phenotypes is presented in Table 3.

The minimum biomarker panel in research should include total 25(OH)D, DBP or free/bioavailable vitamin D where feasible, PTH, calcium, phosphate, magnesium, albumin, renal and liver function, CRP, IL-6, TNF-alpha, BMI, waist circumference, glucose or HbA1c, lipid profile, medication exposure, dietary intake, supplement use, season, latitude, and sunlight exposure [8,10,11]. More advanced studies should include kynurenine/tryptophan ratio, quinolinic acid, kynurenic acid, BDNF, metabolomics, microbiome data, and genetic instruments [73,74].

Stratification should be prespecified. Trials should not simply randomize unselected psychiatric patients to vitamin D versus placebo [10,72]. They should enrich for deficiency or insufficiency, define inflammatory and metabolic phenotypes, account for DBP/free vitamin D, include clinically meaningful outcomes, and monitor adherence and safety [118,119]. Such designs are more likely to reveal whether vitamin D correction modifies symptoms, functioning, cognition, or physical health in specific psychiatric subgroups [65,72].

## 11. Clinical and Public Health Implications

The clinical message is deliberately conservative: correct deficiency and improve nutritional care without converting vitamin D into an unsupported psychiatric remedy. Current guidance argues against indiscriminate population-based screening in asymptomatic nonpregnant adults and highlights uncertainty about optimal 25(OH)D thresholds for disease prevention in people without established indications [1,88]. Psychiatric populations, however, often differ from healthy community samples because they may have medical and behavioral risk factors such as limited sunlight exposure, obesity, pregnancy or lactation, malabsorption, chronic kidney or liver disease, medication burden, severe mental illness, poor diet, and multimorbidity [28,88].

Testing is therefore most defensible when these general medical indications or high-risk features are present: pregnancy or lactation; childhood or adolescent nutritional risk; severe dietary restriction; low outdoor exposure; frailty or falls risk; institutional care; severe mental illness; obesity; antipsychotic-associated metabolic complications; darker skin at high latitude; malabsorption, bariatric surgery, renal or liver disease; or symptoms compatible with deficiency [120,121]. This is not psychiatric screening in isolation; it is integrated clinical nutrition [1,28]. Table 4 translates these principles into a clinical action matrix, distinguishing settings in which vitamin D assessment and correction are defensible from settings where routine psychiatric screening or supplementation is not supported.

Supplementation should be framed as correction of nutritional deficiency and maintenance of adequacy, not as stand-alone psychiatric treatment [1,28,98,120,122]. Doses should follow local guidelines, baseline 25(OH)D status, age, pregnancy or lactation status, body weight, comorbidity, drug exposure, and safety considerations [122,123,124]. For dosing context, prevention-oriented guidelines distinguish routine intake from treatment of confirmed deficiency. The 2024 Endocrine Society guideline advises generally healthy adults younger than 75 years suggests against empiric supplementation above the dietary reference intake—600 IU/day for adults up to 70 years and 800 IU/day for those older than 70 years—while suggesting empiric supplementation in selected groups, including children and adolescents, pregnancy, adults older than 75 years, and adults with high-risk prediabetes. In the trials informing those recommendations, daily equivalent doses ranged from 300 to 2000 IU in children and adolescents, 600–5000 IU in pregnancy, 400–3333 IU in adults older than 75 years, and 842–7543 IU in adults with high-risk prediabetes [1]. Polish 2023 guidance provides more operational prophylactic ranges: 1000–2000 IU/day for adults aged 19–75 years when sunlight exposure is inadequate, 2000–4000 IU/day for adults older than 75 years, and 2000 IU/day during pregnancy and lactation when 25(OH)D testing is not available. Obesity generally requires approximately double the age-adjusted prophylactic dose. For documented deficiency, adult cholecalciferol treatment may use 4000 IU/day or weekly, biweekly, or monthly regimens listed in the guideline [94]. These ranges should be interpreted as general medical and deficiency-correction guidance rather than psychiatric-specific dosing.

Safety matters. Vitamin D toxicity is uncommon but clinically significant, particularly with high-dose, prolonged, unsupervised supplementation [125,126]. Hypercalcemia, hypercalciuria, nephrolithiasis, renal impairment, gastrointestinal symptoms, confusion, and interactions with calcium intake or specific disorders must be considered [8,126,127]. High-dose supplementation in psychiatric patients should not be casual, especially when adherence, cognition, substance use, renal disease, or polypharmacy complicate monitoring [125,128].

In psychiatric populations, monitoring should also account for treatment setting, cognition, adherence, and psychotropic polypharmacy [1,5]. Patients with cognitive impairment, severe negative symptoms, long-term hospitalization, residential care, substance use, or fragmented prescribing may be at risk of duplicated over-the-counter supplementation, unsupervised high-dose use, or unrecognized calcium co-supplementation. In these groups, vitamin D should be documented on the medication chart, supervised when necessary, and reviewed together with calcium-containing preparations. Before high-dose replacement, clinicians should consider baseline 25(OH)D, serum calcium, phosphate, renal function, and, when clinically indicated, PTH; calcium and 25(OH)D should be reassessed after the correction phase or earlier if symptoms of toxicity, renal impairment, nephrolithiasis, hyperparathyroidism, or granulomatous disease are present [129]. Lithium-treated patients require particular caution because lithium may be associated with hypercalcemia and hyperparathyroidism; empirical high-dose vitamin D or combined calcium/vitamin D therapy should be avoided until calcium and PTH status are clarified. Conversely, patients receiving anticonvulsants or antiepileptic mood stabilizers, particularly enzyme-inducing agents, may have increased risk of vitamin D deficiency and bone-health complications and may require more frequent monitoring and guideline-based dose adjustment rather than unmonitored dose escalation [130]. These safeguards are intended to support safe correction of deficiency and do not constitute psychiatric-specific vitamin D dosing recommendations [94,124,125,128,131].

A nutrition-first approach is preferable. Diet quality, fortified foods, safe sunlight exposure, body weight, physical activity, sleep, smoking cessation, cardiometabolic care, and social support should accompany supplementation when indicated [8,120,132,133]. Psychiatric services can integrate vitamin D assessment into broader physical-health pathways, especially for severe mental illness [51,87,133]. The goal is not to medicalize sunlight or prescribe supplements indiscriminately, but to correct modifiable nutritional deficits in patients whose psychiatric illness increases medical vulnerability [28,120].

## 12. Controversies and Limitations

The controversies in this field are not peripheral; they are the field. A rigorous review must treat reverse causality, assay variation, outcome heterogeneity, threshold uncertainty, and supplement enthusiasm as central interpretive hazards rather than as a short limitations paragraph [64,65,117]. The central limitation of the literature is confounding. Low 25(OH)D may reflect limited outdoor activity, poor diet, obesity, socioeconomic adversity, illness severity, hospitalization, institutionalization, season, latitude, skin pigmentation, smoking, alcohol use, physical illness, and medication exposure [11,43,67,129,134]. Depression, psychosis, and severe anxiety can themselves create low vitamin D status [17,71]. Cross-sectional associations are therefore insufficient for causal inference [64,72]. Reverse causality is particularly important in adult psychiatric samples. A patient with negative symptoms may rarely leave home; a depressed patient may stop exercising; an institutionalized patient may have little sunlight exposure; an obese patient may have lower measured 25(OH)D due to volumetric dilution or sequestration [8,10]. Treating low 25(OH)D as a primary cause in these contexts would be clinically naive [8,65]. Measurement is another major limitation. Assays differ, standardization is incomplete, and thresholds vary across organizations and outcomes [73,74,135]. Total 25(OH)D may not adequately represent free or bioavailable vitamin D in pregnancy, liver disease, renal disease, inflammatory states, or across different DBP genotypes [1,75,119]. Psychiatric studies rarely measure DBP, free vitamin D, PTH, calcium, magnesium, renal function, diet, season, or sunlight exposure in sufficient detail [1,75].

Clinical trials have their own weaknesses: short duration, low power, heterogeneous dosing, variable baseline vitamin D status, variable adherence, mixed populations, co-supplementation, unmeasured inflammation, and inconsistent psychiatric endpoints [28,88]. Large trials in generally healthy populations can be under-informative for deficient clinical subgroups, whereas small trials in symptomatic patients can overestimate effects [88,120]. Publication bias and selective outcome reporting remain concerns [1,121].

The field must also resist supplement hype. Vitamin D is biologically important, and deficiency is common in some psychiatric populations, but plausible mechanisms do not equal therapeutic efficacy [28,122]. Overmedicalization can divert attention from social determinants, diet quality, physical activity, sleep, housing, poverty, and evidence-based psychiatric treatment [120,123]. Vitamin D should be studied and used as one component of comprehensive clinical nutrition, not as a substitute for psychiatric care [124,125].

## 13. Future Directions

The next research phase should be narrower, not broader. The field does not need more unselected cross-sectional studies of serum 25(OH)D and symptom scores [64,65,117]. It needs developmentally timed cohorts, deficiency-enriched trials, biomarker panels, individual participant data meta-analyses, and pragmatic clinical nutrition studies embedded in psychiatric care [11,118].

**Figure 4:**
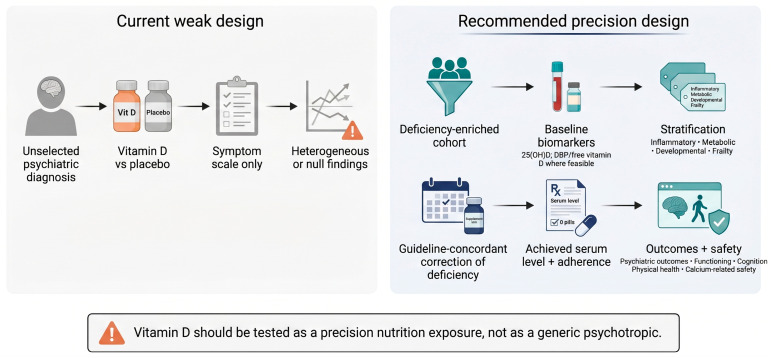
From conventional non-stratified supplementation trials to precision nutrition designs. Conventional vitamin D trials in psychiatry often enroll unselected diagnostic groups, compare vitamin D with placebo, rely mainly on symptom scales, and produce heterogeneous or null findings. A precision nutrition design should enrich for deficiency or insufficiency, measure baseline biomarkers including 25(OH)D and, where feasible, DBP/free vitamin D, stratify participants by inflammatory, metabolic, developmental, or frailty-related phenotypes, correct deficiency in a guideline-concordant manner, confirm achieved serum levels and adherence, and evaluate psychiatric, functional, cognitive, physical health, and calcium-related safety outcomes. Vitamin D should be evaluated as a precision nutrition exposure, not as a generic psychotropic intervention.

Future research should prioritize designs that match biology. Large birth cohorts with maternal samples, cord blood, neonatal dried blood spots, DBP, genetic data, and long-term psychiatric follow-up are essential [17,71]. These cohorts should include repeated exposure measurements, seasonality, diet, supplementation, maternal inflammation, obstetric complications, parental psychiatric history, socioeconomic factors, and polygenic liability [8,10]. The required shift from conventional non-stratified supplementation trials to biomarker-enriched precision nutrition designs is summarized in Figure 4. Together, the evidence-calibration framework in Table 1 and the trial-design schema in Figure 4 are intended to make explicit why unselected supplementation trials are often poorly aligned with the underlying precision hypothesis: they test broad diagnostic categories rather than deficiency-enriched, developmentally timed, inflammatory, metabolic, or frailty-related phenotypes.

Mendelian randomization should be integrated with measured biomarkers and environmental exposures [8,73,74]. Genetic instruments for 25(OH)D and DBP are useful, but psychiatric outcomes are complex, and genetic liability can act through multiple pathways [75]. Triangulation—combining observational cohorts, genetic analyses, sibling comparisons, negative controls, and randomized trials—is more persuasive than any single method [1,119].

Randomized controlled trials should be biomarker-enriched [1,88]. Depression trials should recruit patients with low 25(OH)D and prespecified inflammatory or metabolic phenotypes, use adequate dosing to correct deficiency, monitor serum response, and evaluate remission, functioning, fatigue, cognition, and inflammatory markers [28,88,120]. Psychosis trials should focus on first-episode and high-risk groups with deficiency, include cognition and negative symptoms, and measure cardiometabolic outcomes [1,121]. Severe mental illness studies should test pragmatic nutrition pathways rather than isolated supplementation alone [28,122].

Perinatal trials require particular care. They should examine guideline-concordant supplementation, baseline deficiency, timing from preconception or early pregnancy, maternal DBP/free vitamin D, placental biology, neonatal status, and long-term child neurodevelopment [120,123,124]. Outcomes should include ADHD, ASD traits, cognition, emotion regulation, sleep, motor development, and later psychiatric diagnoses, while avoiding deterministic language and ensuring ethical communication to families [28,125].

Multi-omics approaches can clarify mechanisms. Genomics, epigenomics, transcriptomics, proteomics, metabolomics, microbiome profiling, and immune phenotyping may identify vitamin D-responsive pathways and subgroups [8]. However, omics data must be linked to clinical outcomes and nutritional exposures [8,128]. The aim is not to produce complex biomarker panels without clinical utility, but to identify actionable phenotypes for prevention and treatment [129].

Standardization is urgently needed. Studies should report baseline 25(OH)D, assay method, season, latitude, dose, formulation, adherence, achieved serum levels, DBP or free vitamin D where possible, PTH/calcium safety data, psychiatric outcome definitions, concomitant medications, and adverse events [87]. Individual participant data meta-analyses may help identify effect modifiers that aggregate-level meta-analyses cannot resolve [24,51].

## 14. Evidence-to-Action Framework for Nutritional Psychiatry

**Figure 5:**
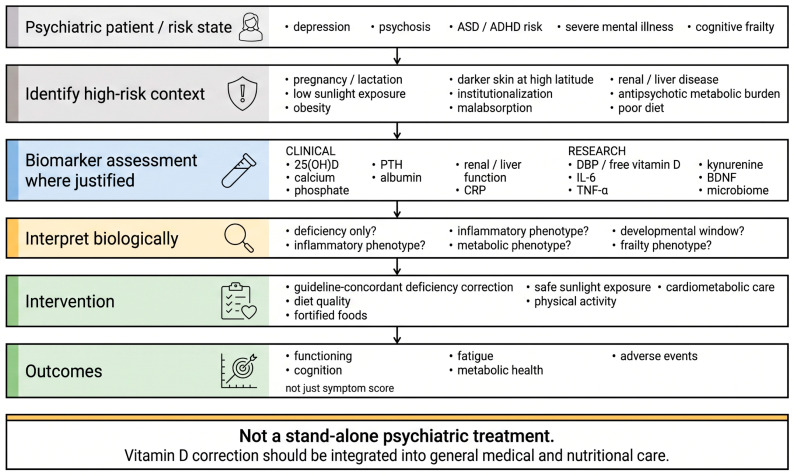
Evidence-to-action framework for vitamin D assessment and correction in psychiatric populations. The framework begins with a psychiatric patient or risk state, followed by identification of high-risk contexts such as pregnancy or lactation, low sunlight exposure, obesity, darker skin pigmentation at high latitude, institutionalization, malabsorption, renal or liver disease, antipsychotic-associated metabolic burden, and poor diet. Biomarker assessment should be clinically justified and may include 25(OH)D, calcium, phosphate, PTH, albumin, renal and liver function, and CRP, with DBP/free vitamin D, inflammatory markers, kynurenine, BDNF, and microbiome measures reserved mainly for research. Interpretation should distinguish nutritional deficiency from inflammatory, metabolic, developmental, or frailty-related phenotypes. Intervention should focus on guideline-concordant deficiency correction, diet quality, fortified foods, safe sunlight exposure, physical activity, and cardiometabolic care. Outcomes should include functioning, cognition, fatigue, metabolic health, adverse events, and calcium-related safety, not psychiatric symptom scores alone. Vitamin D correction should be integrated into general medical and nutritional care and should not be presented as a stand-alone psychiatric treatment.

For clinical nutrition and psychiatry, the practical implication is a staged decision process rather than diagnosis-driven supplementation: identify clinical states in which vitamin D deficiency is likely or clinically consequential; characterize nutritional exposure and vitamin D biology; add inflammatory and metabolic stratification where relevant; correct deficiency in a guideline-concordant manner; and measure outcomes that matter to patients, including functioning, cognition, fatigue, metabolic health, and adverse events. This staged logic is summarized in Figure 5, while a more detailed domain-specific evidence-to-action matrix is provided as Supplementary Table S2.

The proposed staged clinical pathway is summarized in Figure 5, emphasizing that vitamin D assessment and correction should be driven by high-risk context, biomarker interpretation, biological phenotype, and clinically meaningful outcomes rather than by psychiatric diagnosis alone.

This framework also protects against two common errors. The first is therapeutic inflation, in which plausible biology is converted into a claim of psychiatric efficacy before adequate trials exist. The second is therapeutic nihilism, in which negative unselected trials are interpreted as evidence that vitamin D biology is irrelevant. The correct inference lies between these positions: vitamin D is neither a psychiatric panacea nor a trivial correlate. It is a modifiable nutritional exposure whose relevance is conditional on timing, baseline status, and biological phenotype.

## 15. Conclusions

Vitamin D should not be presented as a universal treatment for psychiatric disorders. The randomized evidence does not support such a claim, and overstatement would weaken the credibility of nutritional psychiatry. A more defensible conclusion is that vitamin D biology may be clinically relevant in selected contexts: early neurodevelopmental vulnerability, inflammatory or metabolically complicated depression, psychosis liability, severe mental illness with nutritional deprivation, and frailty or multimorbidity. The appropriate unit of interpretation is therefore phenotype and timing, not diagnosis alone.

Clinically, psychiatrists should not prescribe vitamin D as an antidepressant, antipsychotic, mood stabilizer, or neurodevelopmental treatment. Their role is to identify patients at high risk of deficiency, integrate vitamin D assessment into physical-health care when indicated, and ensure correction of deficiency according to established medical guidance. We do not propose psychiatric-specific dosing regimens. As practical reference points, dietary reference intakes are 600 IU/day for adults aged 19–70 years and 800 IU/day thereafter; Polish guidance supports 1000–2000 IU/day for adults with inadequate sunlight exposure, 2000–4000 IU/day after age 75 years, and 2000 IU/day during pregnancy or lactation when 25(OH)D testing is unavailable, with approximately double prophylactic dosing in obesity. Confirmed adult deficiency may be treated with cholecalciferol 4000 IU/day or guideline-listed intermittent regimens, with follow-up monitoring of 25(OH)D and calcium-related safety.

Operationally, psychiatric diagnosis alone should not trigger routine vitamin D testing or supplementation in otherwise low-risk patients. Targeted assessment and guideline-concordant correction of deficiency are most defensible in five clinical groups: pregnant or lactating patients and other perinatal risk states; children and adolescents with neurodevelopmental vulnerability plus nutritional restriction, low outdoor exposure, obesity, chronic illness, or anticonvulsant exposure; patients with depression and documented or likely deficiency in the context of inflammation, obesity, metabolic syndrome, fatigue, poor diet, or low sunlight exposure; patients with first-episode psychosis or severe mental illness complicated by institutionalization, negative symptoms, antipsychotic-associated metabolic burden, poor diet, smoking, low sunlight exposure, or bone-health risk; and older or cognitively impaired patients with frailty, falls risk, sarcopenia, multimorbidity, renal disease, polypharmacy, or residential care. In all groups, vitamin D management should remain a medical and nutritional intervention: correction should follow local deficiency guidelines, avoid psychiatric-specific dosing assumptions, include calcium-related safety monitoring when high-dose replacement is used, and be integrated with diet quality, physical activity, cardiometabolic care, and evidence-based psychiatric treatment.

Future research should move beyond total 25(OH)D-only models and non-stratified supplementation trials. The field needs developmentally timed cohorts, neonatal biomarkers, DBP/free vitamin D measurement, inflammatory and metabolic phenotyping, Mendelian randomization integrated with environmental exposure data, and pragmatic nutrition trials embedded in psychiatric services. Vitamin D is best positioned not as a simple supplement for mental illness, but as a modifiable nutrient-related signal within lifespan neurodevelopmental, neuroimmune, and immunometabolic risk, with clinical relevance determined by timing, deficiency status, biological phenotype, and comorbidity.

## Figures and Tables

**Table 1:** Evidence-calibration framework and search architecture for this narrative review. The table summarizes how different categories of evidence were prioritized and interpreted. The review distinguishes biological plausibility, developmental risk modulation, adult biomarker association, deficiency correction, psychiatric symptom treatment, and disease prevention. This hierarchy was used to avoid conflating mechanistic plausibility or observational association with proven psychiatric efficacy.

Evidence Layer	Preferred Sources and Study Designs	Permissible Inference	Main Interpretive Constraint
Vitamin D biology and measurement	Endocrine guidelines, clinical nutrition guidance, assay standardization papers, reviews of vitamin D metabolism, DBP/free vitamin D biology, VDR signaling, and 25(OH)D interpretation	Defines vitamin D status as a biological system involving total 25(OH)D, DBP, albumin-bound, free/bioavailable fractions, activation/catabolism, and VDR signaling	Thresholds vary by outcome, assay, population, pregnancy status, inflammatory state, and comorbidity; total 25(OH)D alone may misclassify biologically relevant exposure
Developmental and perinatal risk evidence	Birth cohorts, maternal pregnancy cohorts, cord-blood studies, neonatal dried-blood-spot studies, sibling or registry designs, long-term neurodevelopmental follow-up	Supports temporality and timing-sensitive risk-modulation hypotheses for ASD, ADHD, psychosis liability, and broader neurodevelopmental vulnerability	Long latency, residual confounding, maternal health, seasonality, socioeconomic factors, nutrition, inflammation, and genetic liability limit causal attribution
Genetic triangulation	Mendelian randomization, genome-wide association studies of 25(OH)D/DBP, genetic correlates of neonatal vitamin D biology, polygenic-risk-integrated analyses	Helps test whether genetically proxied vitamin D biology is compatible with causal or risk-modifying hypotheses	Genetic instruments may be weak, context-insensitive, developmentally non-specific, or pleiotropic; MR does not replace measured exposure during critical windows
Adult psychiatric observational evidence	Cross-sectional and longitudinal psychiatric cohorts, population cohorts, severe mental illness cohorts, metabolic and inflammatory phenotype studies	Identifies deficiency prevalence, illness ecology, comorbidity patterns, and candidate phenotype-enrichment strategies	Reverse causality is substantial: psychiatric symptoms may reduce sunlight exposure, diet quality, activity, and preventive care while increasing obesity, medication burden, and inflammation
Intervention and prevention evidence	Randomized controlled trials, meta-analyses, dose–response meta-analyses, pragmatic trials, large universal-prevention studies, adjunctive supplementation trials	Determines whether supplementation corrects deficiency, modifies symptoms, improves physical-health outcomes, or prevents incident psychiatric illness	Trials often include heterogeneous diagnoses, variable baseline vitamin D status, short duration, inconsistent dosing, weak adherence assessment, and limited inflammatory/metabolic stratification
Mechanistic and translational neuroscience	Human biomarker studies, immune/metabolic studies, neurodevelopmental models, experimental VDR/DBP studies, microglial, kynurenine, mitochondrial, HPA-axis, and microbiome research	Establishes biological plausibility and identifies candidate biomarkers, pathways, and stratification targets	Mechanistic plausibility does not establish psychiatric efficacy; peripheral biomarkers may not reflect CNS activity; pathways are transdiagnostic and non-specific
Clinical guidelines and public-health recommendations	Endocrine, nutrition, obstetric, pediatric, geriatric, and preventive-medicine guidance	Defines clinically defensible testing, deficiency correction, safety monitoring, and supplementation boundaries	Guidelines usually address skeletal, endocrine, nutritional, or public-health outcomes rather than psychiatric efficacy; they should not be overextended into unsupported psychiatric indications
Evidence synthesis and clinical interpretation	Systematic reviews, umbrella reviews, meta-analyses, narrative synthesis, evidence-to-action frameworks	Integrates heterogeneous evidence into a lifespan precision-nutrition model distinguishing risk modulation, association, treatment response, and deficiency correction	Pooled estimates can obscure baseline deficiency, achieved serum response, developmental timing, phenotype heterogeneity, dose, duration, seasonality, and comorbidity

Abbreviations: 25(OH)D, 25-hydroxyvitamin D; ADHD, attention-deficit/hyperactivity disorder; ASD, autism spectrum disorder; DBP, vitamin D-binding protein; HPA, hypothalamic–pituitary–adrenal; MR, Mendelian randomization; VDR, vitamin D receptor.

**Table 2:** Evidence-calibrated mechanisms linking vitamin D biology to psychiatric phenotypes.

Mechanistic Domain and Key Markers	Most Relevant Psychiatric Context	Evidence-Calibrated Translational Interpretation
Developmental vitamin D signaling: maternal 25(OH)D, neonatal 25(OH)D, DBP, placental transfer, fetal VDR signaling	Neurodevelopmental vulnerability; ASD/ADHD traits; later psychosis liability	Strongest relevance lies in timing-sensitive developmental risk modulation, not adult symptom treatment. Prioritize pregnancy, cord-blood, neonatal dried-blood-spot, and long-term birth-cohort designs.
VDR signaling and local activation: VDR, CYP27B1, CYP24A1, 1,25(OH)_2_D, neural and immune-cell expression	Broad biological plausibility across neurodevelopment, inflammation, synaptic biology, cognition, and affective phenotypes	Mechanistically credible but clinically non-specific. Interpret as a system-level pathway, not as evidence that total 25(OH)D alone captures psychiatric relevance.
DBP, free, and bioavailable vitamin D biology: GC gene, DBP, albumin, free/bioavailable 25(OH)D	Pregnancy, inflammation, obesity, liver/renal disease, severe mental illness, diverse ancestry groups	Total 25(OH)D may misclassify biologically available vitamin D in key psychiatric populations. Add DBP/free or bioavailable vitamin D in perinatal, psychosis, and inflammatory-depression studies.
Immune regulation and neuroinflammation: CRP, IL-6, TNF-α, innate/adaptive immunity, microglia	Inflammatory depression; psychosis with metabolic comorbidity; suicidality; cognitive impairment with systemic inflammation	Best viewed as a stratification pathway. Trials should enrich for low 25(OH)D plus inflammatory phenotype rather than broad DSM/ICD diagnosis alone.
Tryptophan–kynurenine/NMDA pathway: IDO/TDO, tryptophan, kynurenine, kynurenic acid, quinolinic acid, NMDA signaling	Depression with fatigue or inflammation; psychosis; suicidality; cognitive dysfunction	Psychiatric relevance is strong, but vitamin D-specific causality remains uncertain. Use kynurenine markers as part of multi-biomarker panels, not as stand-alone surrogate endpoints.
Monoaminergic, glutamatergic, and synaptic pathways: dopamine, serotonin, glutamate/NMDA balance, BDNF, synaptic plasticity, myelination	ADHD traits; psychosis liability; affective symptoms; negative symptoms; cognition	Mostly mechanistic and preclinical. Avoid simplified claims that vitamin D “corrects neurotransmitters”; test these pathways in developmental and high-risk cohorts.
Oxidative stress and mitochondrial bioenergetics: ROS, antioxidant pathways, mitochondrial respiration, cellular energetics	Fatigue-dominant depression; bipolar disorder; psychosis; severe mental illness with metabolic syndrome	Biologically plausible but clinically non-specific. Most relevant in metabolically vulnerable or inflamed patients, ideally within broader nutritional and cardiometabolic interventions.
HPA-axis, circadian, and stress-system regulation: cortisol, glucocorticoid signaling, sleep–circadian disruption, inflammatory stress response	Stress-related depression; anxiety traits; adolescent vulnerability; perinatal stress	Hypothesis-generating pathway. Future studies should measure seasonality, sleep, stress exposure, inflammation, and sunlight exposure to reduce reverse-causality bias.
Gut–microbiota–immune–brain axis: gut barrier function, microbiota, microbial metabolites, mucosal immunity, diet quality	Transdiagnostic nutritional vulnerability; depression with metabolic dysfunction; ASD-related traits; severe mental illness	Emerging translational pathway. Vitamin D should be studied as part of broader nutritional ecology, not as an isolated supplement exposure.

Abbreviations: 25(OH)D, 25-hydroxyvitamin D; 1,25(OH)_2_D, 1,25-dihydroxyvitamin D; ADHD, attention-deficit/hyperactivity disorder; ASD, autism spectrum disorder; BDNF, brain-derived neurotrophic factor; CRP, *C*-reactive protein; DBP, vitamin D-binding protein; GC, group-specific component gene; HPA, hypothalamic–pituitary–adrenal; IDO, indoleamine 2,3-dioxygenase; IL-6, interleukin-6; NMDA, *N*-methyl-D-aspartate; ROS, reactive oxygen species; TDO, tryptophan 2,3-dioxygenase; TNF-α, tumor necrosis factor-alpha; VDR, vitamin D receptor.

**Table 3:** Biomarker-informed precision framework for vitamin D in nutritional psychiatry. The table identifies clinical and developmental contexts in which vitamin D biology is most likely to be informative and links each phenotype with relevant biomarkers, interpretation, and preferred clinical or research endpoints. It is intended to support stratified research design and clinically sensible nutritional assessment.

Precision Phenotype/Enrichment Context	Context-Specific Biomarker Emphasis	Actionable Interpretation	Preferred Clinical or Research Endpoint
Inflammatory/metabolic depression	CRP, IL-6, TNF-α, HbA1c, lipids, BMI/waist; research add-ons: DBP/free 25(OH)D, kynurenine/tryptophan ratio	Vitamin D is best treated as an adjunctive nutritional hypothesis when deficiency coexists with inflammation, adiposity, poor diet, or metabolic risk. This phenotype is suitable for deficiency- and inflammation-enriched clinical trials, but current evidence does not establish vitamin D as an antidepressant intervention.	High priority: deficiency- and inflammation-enriched RCTs measuring remission, fatigue, function, inflammatory markers, achieved 25(OH)D, adherence, and adverse events
Perinatal/neonatal developmental risk	Preconception or early-pregnancy 25(OH)D, DBP/free vitamin D, PTH/calcium, maternal inflammation, cord or neonatal 25(OH)D/DBP, season, diet, genetic liability	This is the most timing-sensitive phenotype. Vitamin D should be interpreted as a developmental exposure and risk-modifying signal, not as a deterministic cause of later psychiatric illness.	Very high priority: birth cohorts and long-term trials with ASD/ADHD traits, cognition, emotional regulation, sleep, motor development, and later psychiatric outcomes
Neurodevelopmental vulnerability: ADHD/ASD traits	Early-life 25(OH)D, DBP, diet quality, inflammatory markers, microbiome, perinatal risk factors, polygenic liability	Current evidence supports probabilistic risk modulation. Correct deficiency and ensure nutritional adequacy, but avoid claims that vitamin D prevents or treats ADHD/ASD.	High priority: longitudinal developmental cohorts and targeted trials in deficient or biologically enriched children, focused on trajectories rather than binary diagnosis alone
Clinical high risk/first-episode psychosis	25(OH)D, DBP/free 25(OH)D, CRP, metabolic profile, BMI/waist, cognition, negative symptoms, antipsychotic exposure	Low vitamin D is common and clinically relevant, but psychiatric symptom efficacy remains unproven. The main value is nutritional, metabolic, bone-health, and physical-health risk management.	Moderate–high priority: pragmatic early-psychosis studies assessing deficiency correction, metabolic health, cognition, negative symptoms, function, adherence, and safety
Severe mental illness with nutritional deprivation	Calcium/PTH, metabolic profile, diet quality, food insecurity, institutionalization, medication burden, smoking, physical activity, sunlight exposure	Vitamin D status is a marker of physical-health inequality and nutritional vulnerability. Management should be embedded in a clinical nutrition pathway rather than isolated supplementation.	High priority: implementation studies in inpatient, community, and long-term care settings; endpoints should include deficiency correction, cardiometabolic health, falls, bone health, and service-level uptake
Metabolic syndrome-associated psychiatric illness	HbA1c, fasting glucose/insulin resistance, lipids, liver enzymes, CRP, waist circumference, sleep apnea risk, antipsychotic or mood-stabilizer exposure	Obesity and inflammation can alter vitamin D status and psychiatric morbidity. The intervention model should be metabolic and nutritional, not supplement-only.	Moderate–high priority: combined diet, weight, activity, cardiometabolic, and deficiency-correction studies with mood, fatigue, metabolic, and inflammatory endpoints
Older-age cognitive frailty	Frailty score, falls history, gait speed, grip strength, cognition, albumin, renal function, polypharmacy, sarcopenia markers	Vitamin D is best viewed as one component of frailty, musculoskeletal vulnerability, cognition, and multimorbidity care, not as a cognitive enhancer.	Moderate priority: multimodal geriatric trials assessing falls, function, cognition, institutionalization risk, muscle outcomes, safety, and quality of life

Abbreviations: 25(OH)D, 25-hydroxyvitamin D; ADHD, attention-deficit/hyperactivity disorder; ASD, autism spectrum disorder; BMI, body mass index; CRP, *C*-reactive protein; DBP, vitamin D-binding protein; HbA1c, glycated hemoglobin; IL-6, interleukin-6; PTH, parathyroid hormone; RCT, randomized controlled trial; TNF-α, tumor necrosis factor-alpha.

**Table 4:** Clinical action matrix for vitamin D assessment and correction in psychiatric and nutritional settings. The matrix identifies settings in which assessment is clinically defensible, summarizes reasonable actions, and defines boundary conditions for practice and research. Decisions should be individualized according to deficiency risk, comorbidity, developmental stage, diet, sunlight exposure, safety, and local guidance.

Clinical Context	When Vitamin D Assessment Is Most Defensible	Clinically Reasonable Action	Boundary Condition/Research Priority
Pregnancy and perinatal care	Deficiency risk, limited sunlight exposure, darker skin at high latitude, obesity, dietary restriction, malabsorption, prior deficiency, or other antenatal nutritional risk	Ensure guideline-concordant prenatal nutrition; assess and correct deficiency when clinically indicated; integrate vitamin D with broader maternal nutrition, inflammation, and fetal-development risk assessment	Do not claim that supplementation alone prevents ASD, ADHD, or schizophrenia. Priority: preconception/early-pregnancy cohorts, cord/neonatal biomarkers, DBP/free vitamin D, and long-term neurodevelopmental follow-up.
Children and adolescents with neurodevelopmental vulnerability	Dietary selectivity, restricted diets, low outdoor activity, obesity, chronic illness, anticonvulsant exposure, ASD/ADHD traits with nutritional risk, or documented deficiency	Perform nutritional assessment; correct confirmed deficiency; support diet quality, fortified foods where appropriate, safe outdoor activity, and family-centered nutritional care	Do not attribute neurodevelopmental disorders to vitamin D deficiency. Priority: longitudinal cohorts integrating diet, sunlight exposure, DBP, inflammatory markers, microbiome, genetics, and dimensional developmental outcomes.
Depression with deficiency, inflammation, or metabolic risk	Depression coexisting with low sunlight exposure, obesity, poor diet, fatigue, elevated CRP, diabetes/prediabetes, metabolic syndrome, pregnancy/postpartum status, or recurrent deficiency	Correct deficiency according to clinical guidance; address diet, weight, activity, sleep, and cardiometabolic risk; consider vitamin D as an adjunctive nutritional target only in biologically enriched patients	Do not use vitamin D as antidepressant monotherapy or universal prevention. Priority: deficiency- and inflammation-enriched RCTs measuring remission, fatigue, function, inflammatory markers, achieved 25(OH)D, adherence, and safety.
First-episode psychosis and severe mental illness	Indoor lifestyle, negative symptoms, institutionalization, antipsychotic-associated weight gain, poor diet, smoking, low preventive care access, metabolic syndrome, bone-health risk, or previous deficiency	Screen when clinically justified; correct deficiency; integrate with physical-health monitoring, metabolic care, dietetic input, exercise support, bone-health assessment, and social interventions	Do not assume supplementation improves psychotic symptoms. Priority: pragmatic trials and implementation studies using physical-health, metabolic, cognitive, functional, falls, bone-health, and adherence outcomes.
Older adults with cognitive frailty or functional decline	Frailty, falls risk, sarcopenia, low sunlight exposure, institutional care, multimorbidity, renal disease, poor nutrition, polypharmacy, or cognitive symptoms with nutritional vulnerability	Individualize testing and supplementation; correct deficiency cautiously; monitor calcium/PTH/renal function when indicated; combine with falls prevention, resistance exercise, protein adequacy, medication review, and geriatric care	Do not use high-dose empirical therapy without monitoring or frame vitamin D as a cognitive enhancer. Priority: multimodal geriatric trials assessing falls, function, cognition, institutionalization, muscle outcomes, quality of life, and toxicity.
Generally healthy adults without risk factors	Usually not indicated for psychiatric reasons alone; consider only if general medical risk factors for deficiency are present	Follow dietary reference intakes, public-health guidance, and standard clinical indications rather than psychiatric screening	Avoid routine 25(OH)D screening or high-dose supplementation for psychiatric prevention. Priority: population prevention claims require stronger evidence and should distinguish deficiency correction from disease prevention.

Abbreviati-ons: 25(OH)D, 25-hydroxyvitamin D; ADHD, attention-deficit/hyperactivity disorder; ASD, autism spectrum disorder; CRP, *C*-reactive protein; DBP, vitamin D-binding protein; PTH, parathyroid hormone; RCT, randomized controlled trial.

## Data Availability

No new data were created or analyzed in this study. Data sharing is not applicable to this article.

## Supplementary Materials

The following supporting information can be downloaded at https://www.mdpi.com/article/10.3390/nu18121877/s1. Supplementary Table S1: Key quantitative findings from pivotal randomized and developmental vitamin D studies relevant to psychiatry; Supplementary Table S2: Evidence-to-action matrix distinguishing developmental risk, biomarker association, deficiency correction, and psychiatric treatment evidence for vitamin D across psychiatric domains. References [18,23,24,54] are cited in the Supplementary Materials.

## Abbreviations

The following abbreviations are used in this manuscript:

1,25(OH)_2_D	1,25-dihydroxyvitamin D
25(OH)D	25-hydroxyvitamin D
ADHD	attention-deficit/hyperactivity disorder
AI	artificial intelligence
ASD	autism spectrum disorder
BDNF	brain-derived neurotrophic factor
BMI	body mass index
CC BY	Creative Commons Attribution license
CI	confidence interval
CNS	central nervous system
COPSAC	Copenhagen Prospective Studies on Asthma in Childhood
COPYCH	Copenhagen Prospective Study on Neuro-PSYCHiatric Development
CRP	*C*-reactive protein
CYP2R1	cytochrome P450 family 2 subfamily R member 1
CYP24A1	cytochrome P450 family 24 subfamily A member 1
CYP27B1	cytochrome P450 family 27 subfamily B member 1
DBP	vitamin D-binding protein
DFEND	randomized clinical trial of vitamin D supplementation in early psychosis
DHCR7	7-dehydrocholesterol reductase
DSM	Diagnostic and Statistical Manual of Mental Disorders
GC	group-specific component gene
HbA1c	glycated hemoglobin
HPA	hypothalamic–pituitary–adrenal
ICD	International Classification of Diseases
IDO	indoleamine 2,3-dioxygenase
IL-6	interleukin-6
IU	international unit
MEDLINE	Medical Literature Analysis and Retrieval System Online
MR	Mendelian randomization
NADSYN1	NAD synthetase 1
NMDA	*N*-methyl-D-aspartate
OR	odds ratio
PANSS	Positive and Negative Syndrome Scale
PHQ-8	Patient Health Questionnaire-8
PRISMA	Preferred Reporting Items for Systematic Reviews and Meta-Analyses
PTH	parathyroid hormone
RCT	randomized controlled trial
ROS	reactive oxygen species
TDO	tryptophan 2,3-dioxygenase
TNF-α	tumor necrosis factor-alpha
UVB	ultraviolet B
VDR	vitamin D receptor
VITAL	Vitamin D and Omega-3 Trial
VITAL-DEP	Vitamin D and Omega-3 Trial-Depression Endpoint Prevention
Vitamin D_2_	ergocalciferol
Vitamin D_3_	cholecalciferol
