# Supplementary material for: Vitamin D as a Lifespan Neuroimmune Signal in Psychiatry: From Developmental Risk to Precision Nutrition
Source: Nutrients. 2026 Jun 10;18(12):1877. doi: 10.3390/nu18121877 (PMC13306076; doi:10.3390/nu18121877)
Supplement: Supplementary file 1 [file nutrients-18-01877-s001.zip › Supplementary Table S1.pdf]

Supplementary Table S1: Key quantitative findings from pivotal randomized and developmental vitamin D studies relevant to psychiatry

Study	Design and vitamin D contrast	25(OH)D status	Main quantitative finding and interpretation
VITAL-DEP [18]	Prevention RCT; 18,353 adults ≥50 years without clinically relevant depressive symptoms; vitamin D3 2000 IU/day vs placebo; median follow-up 5.3 years	Mean baseline 25(OH)D in the parent trial: 30.8 ng/mL, indicating a generally sufficient rather than deficiency-enriched population	Depression or clinically relevant depressive symptoms: 609 vs 625 events; HR 0.97, 95% CI 0.87–1.09; P = .62. PHQ-8 change: MD 0.01, 95% CI −0.04 to 0.05. These data argue against universal depression prevention in generally sufficient older adults, but do not exclude benefit in deficient or biologically enriched subgroups.
DFEND [23]	Adjunctive-treatment RCT; 149 adults with early psychosis; cholecalciferol 120,000 IU monthly vs placebo; 6 months	Baseline 25(OH)D: 14.30 vs 15.93 ng/mL; 74.6% had <20 ng/mL. At 6 months: 32.97 vs 15.89 ng/mL	PANSS total at 6 months: MD 3.57, 95% CI −1.11 to 8.25; P = .13. Vitamin D corrected biochemical deficiency but did not improve psychiatric outcomes, supporting deficiency correction for general health rather than antipsychotic efficacy.
COPSAC/COPYCH [54]	Pregnancy RCT; 623 mothers randomized from gestational week 24 to 1 week postpartum; vitamin D3 2800 IU/day vs 400 IU/day; offspring assessed at age 10	Maternal 25(OH)D measured preintervention and analyzed as a developmental exposure marker	High-dose supplementation was not associated with lower autism or ADHD risk. Higher preintervention 25(OH)D was associated with lower autism risk: OR per 10 nmol/L 0.76, 95% CI 0.59–0.97; P = .034; lower autistic symptom load: β −0.03, 95% CI −0.05 to 0.00; P = .024; and lower ADHD diagnosis risk: OR 0.88, 95% CI 0.78–0.99; P = .033. This supports timing and baseline-status hypotheses rather than a simple supplementation effect.
Ghaemi et al. [24]	Dose-response meta-analysis of 31 RCTs including 24,189 adults; vitamin D3 supplementation across variable doses and durations	Baseline 25(OH)D varied; studies were not uniformly deficiency-enriched	Each additional 1000 IU/day was associated with lower depressive symptoms: SMD −0.32, 95% CI −0.43 to −0.22. The effect was larger in participants with depressive symptoms: SMD −0.57, 95% CI −0.69 to −0.44. Findings suggest a possible short-term symptom signal, but heterogeneity and variable baseline status limit clinical generalization.

Footnote:

25(OH)D, 25-hydroxyvitamin D; RCT, randomized controlled trial; HR, hazard ratio; CI, confidence interval; MD, mean difference; SMD, standardized mean difference; PHQ-8, 8-item Patient Health Questionnaire; PANSS, Positive and Negative Syndrome Scale; ASD, autism spectrum disorder; ADHD, attention-deficit/hyperactivity disorder. To convert 25(OH)D from ng/mL to nmol/L, multiply by 2.5.

References:

1. Okereke OI, Reynolds CF 3rd, Mischoulon D, Chang G, Vyas CM, Cook NR, Weinberg A, Bubes V, Copeland T, Friedenberg G, Lee IM, Buring JE, Manson JE. Effect of Long-term Vitamin D3 Supplementation vs Placebo on Risk of Depression or Clinically Relevant Depressive Symptoms and on Change in Mood Scores: A Randomized Clinical Trial. JAMA. 2020 Aug 4;324(5):471-480. doi: 10.1001/jama.2020.10224.
2. Gaughran F, Stringer D, Wojewodka G, Landau S, Smith S, Gardner-Sood P, Taylor D, Jordan H, Whiskey E, Krivoy A, Ciufolini S, Stubbs B, Casetta C, Williams J, Moore S, Allen L, Rathod S, Boardman A, Khalifa R, Firdosi M, McGuire P, Berk M, McGrath J. Effect of Vitamin D Supplementation on Outcomes in People With Early Psychosis: The DFEND Randomized Clinical Trial. JAMA Netw Open. 2021 Dec 1;4(12):e2140858. doi: 10.1001/jamanetworkopen.2021.40858.
3. Aagaard K, Møllegaard Jepsen JR, Sevelsted A, Horner D, Vinding R, Rosenberg JB, Brustad N, Eliasen A, Mohammadzadeh P, Følsgaard N, Hernández-Lorca M, Fagerlund B, Glenthøj BY, Rasmussen MA, Bilenberg N, Stokholm J, Bønnelykke K, Ebdrup BH, Chawes B. High-dose vitamin D3 supplementation in pregnancy and risk of neurodevelopmental disorders in the children at age 10: A randomized clinical trial. Am J Clin Nutr. 2024 Feb;119(2):362-370. doi: 10.1016/j.ajcnut.2023.12.002.
4. Ghaemi S, Zeraattalab-Motlagh S, Jayedi A, Shab-Bidar S. The effect of vitamin D supplementation on depression: a systematic review and dose-response meta-analysis of randomized controlled trials. Psychol Med. 2024 Nov;54(15):3999-4008. doi: 10.1017/S0033291724001697.
