# Supplementary material for: Vitamin D as a Lifespan Neuroimmune Signal in Psychiatry: From Developmental Risk to Precision Nutrition
Source: Nutrients. 2026 Jun 10;18(12):1877. doi: 10.3390/nu18121877 (PMC13306076; doi:10.3390/nu18121877)
Supplement: Supplementary file 1 [file nutrients-18-01877-s001.zip › Supplementary Table S2.pdf]

Supplementary Table S2. Evidence-to-action matrix distinguishing developmental risk, biomarker association, deficiency correction, and psychiatric treatment evidence for vitamin D across psychiatric domains.

Psychiatric domain / phenotype	Dominant evidence pattern	Strongest defensible inference	Clinical and research implication
Depressive disorders: symptomatic, deficient, inflammatory/metabolic subgroup vs universal prevention	Observational associations are common; small and heterogeneous RCTs suggest possible short-term symptom benefit; large universal-prevention data in generally sufficient older adults are negative.	Vitamin D is not a stand-alone antidepressant. The most plausible signal is adjunctive benefit or biological relevance in deficient, inflamed, obese, nutritionally compromised, or medically comorbid subgroups.	Correct deficiency according to general medical indications. Future trials should enrich for low 25(OH)D, inflammatory phenotype, metabolic vulnerability, seasonality, and achieved serum response rather than recruit unselected depression samples.
Psychosis: developmental liability vs established illness supplementation	Neonatal and early-life studies support risk modulation; adult psychosis cohorts often show low 25(OH)D; early psychosis supplementation trials have not shown clear psychiatric benefit.	The strongest signal is developmental and risk-oriented rather than therapeutic in established psychosis. Adult deficiency remains clinically important as a marker of nutritional and physical-health vulnerability.	Separate perinatal prevention hypotheses from adult treatment hypotheses. In psychosis services, vitamin D assessment belongs within physical-health, metabolic, nutritional, bone-health, and lifestyle pathways.
ADHD and neurodevelopmental traits	Maternal/neonatal cohort studies and systematic reviews report associations between low early-life vitamin D and ADHD risk or traits; genetic and biomarker data are emerging.	Evidence supports probabilistic risk modulation, not deterministic causation. Timing of exposure is likely more important than later supplementation after symptom onset.	Prioritize pregnancy, neonatal, and childhood longitudinal designs with baseline vitamin D status, DBP/free vitamin D, maternal inflammation, socioeconomic factors, and polygenic liability. Avoid prevention claims until trial evidence is stronger.
Autism spectrum disorder and related	Early-life observational studies report associations;	Vitamin D may be part of a broader developmental	Maintain guideline-concordant nutritional adequacy in pregnancy

developmental vulnerability	pediatric supplementation trials are inconsistent; pregnancy high-dose supplementation follow-up has not shown clear reduction in ASD/ADHD risk.	risk architecture, but current evidence does not justify causal or preventive claims for ASD.	and childhood. Future studies should focus on timing, deficiency enrichment, maternal–placental biology, neonatal biomarkers, and long-term developmental phenotyping.
Bipolar disorder and mood instability	Evidence is mainly observational, with heterogeneous links to vitamin D deficiency, illness phase, inflammation, seasonality, and metabolic status; RCT evidence is sparse.	Vitamin D deficiency may mark lifestyle disruption, metabolic burden, or seasonal vulnerability, but a mood-stabilizing effect is unproven.	Treat deficiency for general health. Research should focus on stratified bipolar cohorts with inflammatory/metabolic profiling, medication exposure, seasonality, sleep–circadian measures, and relapse-relevant outcomes.
Anxiety and stress-related phenotypes	Evidence comes from secondary endpoints, small trials, and observational studies; findings are inconsistent and phenotype definitions are often weak.	Vitamin D should not be interpreted as a primary anxiolytic intervention. Associations may reflect sleep disruption, low outdoor activity, stress exposure, inflammation, or comorbidity.	Do not use vitamin D as primary anxiety treatment. Future studies should measure sunlight exposure, sleep, seasonality, HPA-axis markers, inflammatory status, and comorbid depression.
Suicidality	Observational syntheses report associations between low vitamin D and suicidal ideation or attempts, usually in clinically complex populations.	Low vitamin D may identify a high-vulnerability state, but it is not evidence of an anti-suicide effect. Confounding by depression severity, pain, inflammation, deprivation, physical illness, and social adversity is substantial.	Do not frame supplementation as suicide prevention. In suicidal patients, low vitamin D should trigger broader assessment of nutrition, inflammation, physical illness, social deprivation, sleep, pain, and psychiatric acuity.
Cognition, frailty, and later-life psychiatric presentations	Observational associations exist between low vitamin D, cognition, frailty, falls, and functional	Vitamin D is more defensible as a marker and modifiable component of	Correct deficiency within multimodal geriatric care, including falls prevention, resistance exercise, protein adequacy,

	decline; supplementation does not reliably prevent cognitive decline in generally replete older adults.	frailty-related vulnerability than as a cognitive enhancer.	medication review, vascular risk reduction, sleep, and social support.
Severe mental illness: nutritional deprivation and cardiometabolic risk phenotype	Cross-sectional cohorts and clinical audits show high prevalence of deficiency or insufficiency, often linked to indoor living, obesity, poor diet, smoking, institutionalization, antipsychotic exposure, and low preventive care access.	Vitamin D status is a clinically relevant marker of physical-health inequality and nutritional risk in severe mental illness, but psychiatric symptom efficacy is unproven.	Integrate vitamin D assessment and correction into severe mental illness physical-health pathways, especially where obesity, metabolic syndrome, institutionalization, low sunlight exposure, or poor diet are present.

Abbreviations: 25(OH)D, 25-hydroxyvitamin D; ADHD, attention-deficit/hyperactivity disorder; ASD, autism spectrum disorder; DBP, vitamin D-binding protein; HPA, hypothalamic–pituitary–adrenal; RCT, randomized controlled trial.
